# Oxygen supersaturation has negligible effects on warming tolerance across diverse aquatic ectotherms

**DOI:** 10.1371/journal.pbio.3003413

**Published:** 2025-11-04

**Authors:** Graham D. Raby, Jeremy De Bonville, Leroy Reynolds, Zoe Storm, Zara-Louise Cowan, Moa Metz, Anna H. Andreassen, Leon Pfeufer, Emily R. Lechner, Erin M. C. Stewart, Robine H. J. Leeuwis, Rasmus Ern, Lorena Silva-Garay, Michael R. Skeeles, Dominique G. Roche, Rachael Morgan, Leon Green, Ben Speers-Roesch, Suzanne C. Mills, Timothy D. Clark, Fredrik Jutfelt

**Affiliations:** 1 Department of Biology, Trent University, Peterborough, Ontario, Canada; 2 Groupe de recherche interuniversitaire en limnologie, Department of Biological Sciences, Université de Montréal, Montréal, Québec, Canada; 3 PSL Research University: EPHE-UPVD-CNRS, USR 3278 CRIOBE, Papetoai, Moorea, French Polynesia; 4 College of Science and Engineering, James Cook University, Townsville, Queensland, Australia; 5 Department of Biology, Faculty of Natural Sciences, Norwegian University of Science and Technology, Trondheim, Norway; 6 Department of Biological and Environmental Sciences, Faculty of Natural Sciences, University of Gothenburg, Kristineberg Center, Fiskebäckskil, Sweden; 7 National Institute of Aquatic Resources, Technical University of Denmark, Lyngby, Denmark; 8 Department of Biological and Environmental Sciences, Faculty of Natural Sciences, University of Gothenburg, Gothenburg, Sweden; 9 Environmental and Life Sciences Graduate Program, Trent University, Peterborough, Ontario, Canada; 10 School of Life and Environmental Sciences, Deakin University, Geelong, Victoria, Australia; 11 Department of Biology, Carleton University, Ottawa, Ontario, Canada; 12 Institute of Biology, Université de Neuchâtel, Neuchâtel, Switzerland; 13 Department of Biological Sciences, University of Bergen, Bergen, Norway; 14 Department of Biological Sciences, University of New Brunswick, Saint John, New Brunswick, Canada; 15 Laboratoire d’Excellence ‘CORAIL’, France; 16 Institut Universitaire de France, Ministère de l’Enseignement supérieur, de la Recherche et de l’Innovation, 1 rue Descartes, Paris, France; Victoria University of Wellington Faculty of Science, NEW ZEALAND

## Abstract

Under the midday sun, when photosynthesizers are producing oxygen, shallow aquatic ecosystems can become supersaturated with oxygen (>100% air saturation) while they simultaneously peak in water temperature. It has been suggested that oxygen supersaturation could protect water-breathing animals from mortality during heatwaves because of the potential role of oxygen in governing thermal tolerance. Here, we conducted a circumglobal assessment of the effects of ecologically relevant oxygen supersaturation (150%, hyperoxia) on warming tolerance (here, measured using critical thermal maximum, CT_max_) in 14 aquatic ectotherms from diverse marine and freshwater environments (10 fishes, four decapod crustaceans), in a series of 24 experiments that included 147 CT_max_ trials and 1,451 animals using two warming rates (0.3°C min^−1^ and 1°C h^−1^). In 10 of 14 species, there was no effect of oxygen supersaturation relative to normoxic controls. In four species (two tropical reef fishes and two marine decapod crustaceans), we found mixed evidence for effects of oxygen saturation, with most of the effects being small (*ca*. 0.2°C–0.3°C). Thus, contrary to predictions, we conclude that oxygen supersaturation is unlikely to protect most water-breathers from heatwaves and therefore few species distribution models or climate risk assessments will benefit from incorporating oxygen supersaturation.

## Abstract 2

Oxygen supersaturation in aquatic ecosystems has been proposed to buffer against heat stress in water-breathing animals. This study re-examines this question in a diverse panel of aquatic ectotherms, showing that hyperoxia has minimal impact on thermal tolerance.

## Introduction

Shallow aquatic environments are among the most extreme and variable on the planet. The abiotic conditions in tidal pools, reef flats, salt marshes, shallow lake habitats, and streams can change rapidly due to events such as tidal cycles, floods, and phytoplankton blooms. Moreover, climate change and the associated increase in heatwaves are amplifying acute heat stress in many of these aquatic ecosystems, threatening the performance and persistence of resident animals [1].

During daytime, when water temperatures are typically peaking and, in some cases, threatening aquatic animals via heat stress [2], many photosynthetic organisms also reach peak photosynthesis and oxygen production [3]. As a result, oxygen supersaturation (hyperoxia, i.e., dissolved oxygen partial pressures >100% air saturation) regularly occurs in shallow water bodies, commonly reaching levels around 150% of air saturation [3–5] (S1 Table). A leading hypothesis in climate change biology is that the warming tolerance of fish and other ectotherms is limited by oxygen transport capacity [2,6–8]. The “oxygen-limitation” hypothesis proposes that warming creates a mismatch between the temperature-induced rise in metabolic oxygen demand and the capacity of the cardiorespiratory system to supply tissues with oxygen, causing tissue hypoxia and ultimately loss of vital functions [2,6,7]. The simultaneous peaks in temperature and oxygen in shallow water environments give rise to the possibility that natural daily cycles in oxygen could help to protect water-breathing ectotherms by increasing oxygen supply and, in turn, enable the maintenance of performance or survival during periods of high temperature [3].

Relatively few studies have tested the effect of hyperoxia on warming tolerance in aquatic animals, but some data exist. McArley and colleagues [9] reviewed experiments on fish and reported benefits of hyperoxia for warming tolerance in 9 of 20 species tested (also see [10,11]). The mean improvement in critical thermal maximum (CT_max_) across those studies was *ca*. 0.90°C (at 140%–200% air saturation) relative to normoxic controls (i.e., ~100% air saturation) [9], bearing in mind that hyperoxia can become detrimental to fishes when oxygen levels approach 200% [12]. Notably, sample sizes were usually small at 8–10 animals per treatment [9] and typically with only *n* = 1 replicate CT_max_ trial. Some data on aquatic invertebrates have been reported as well. In nymphs of the mayfly *Seratella ignita* exposed to hyperoxia (~285% air saturation), a 1.2°C increase in CT_max_ occurred relative to normoxia, but no significant difference was reported for the nymphs of *Ephemera danica* [13]. While inconsistent and small effects of hyperoxia on warming tolerance suggest a nuanced rather than universal benefit to aquatic animals, a study by Giomi and colleagues [3] stands out as reporting the largest and clearest effects. During a 2°C h^−1^ warming experiment, hyperoxia (140% air saturation) increased warming tolerance by an average of 2.25°C (range 1.2°C–3.5°C) across six marine species from the Red Sea (two fishes, four invertebrates, [3]). All six species live in tropical coastal habitats where oxygen supersaturation and rising sea temperature exhibit similar diurnal cycles, and thus the authors concluded that naturally occurring hyperoxia can protect aquatic animals during heatwaves [3]. Thus, conflicting results across a relatively limited body of evidence highlight the need for a large-scale empirical assessment of whether warming tolerance is limited by oxygen (and by how much), using consistent methods and a broad array of species.

**Fig 1:**
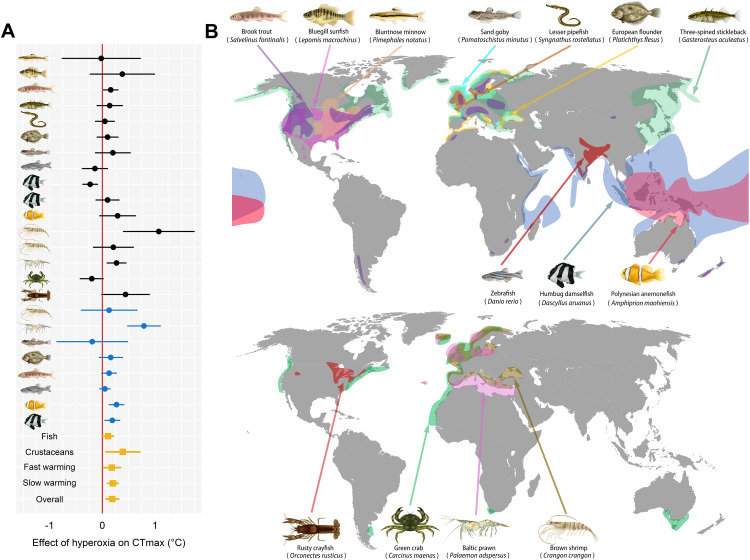
Effect of hyperoxia (150% air saturation) on warming tolerance in 14 aquatic ectotherms from across the globe. **A**: Forest plot showing effect sizes (model estimates ± 95% confidence intervals) for the effect of hyperoxia on warming tolerance. Black symbols are the fast warming (0.3°C min^−1^) trials, blue symbols are the slow warming (1°C h^−1^) trials, and yellow symbols are for a net combined effect with random effects for subgroups of the 24 experiments. The effects were considered statistically significant where the 95% confidence interval does not cross the red vertical line (full statistics given in S3 Table). **B**: Approximate geographical distributions for the 10 species of fish (top) and four species of decapod crustaceans (bottom) used in the laboratory experiments to assess the effects of hyperoxia on upper thermal tolerance (species distributions from aquamaps.org). The map base layer is from SlideLizard (Johanna Liang; https://slidelizard.com/en/blog/powerpoint-world-map). Raw data and analysis code used to estimate the effect sizes are archived on figshare: https://doi.org/10.6084/m9.figshare.30043432.

Here, we assessed the universality of the potential benefit of naturally occurring oxygen supersaturation among marine and freshwater ectotherms via a multi-lab and multi-continental investigation. To do so, we assessed the effect of hyperoxia (150% air saturation) on the warming tolerance of 14 species of aquatic ectotherms. The 14 species included 10 fishes and four decapod crustaceans from a variety of shallow temperate and tropical aquatic habitats (e.g., tide pools and the shallow areas of coral reefs, lakes, rivers, and streams) at varying latitudes, each of which are likely to exhibit oxygen supersaturation similar to the levels used here (Figs 1 and S2, and S1 Table). Warming tolerance was assessed using CT_max_ trials (the temperature at which loss of motor function occurs during acute warming) at the recommended warming rate of 0.3°C min^−1^ [14]. The ecological relevance of CT_max_ has been questioned [15] because warming of 0.3°C min^−1^ is unlikely to occur in nature and because many species will exhibit a total loss of fitness with chronic exposure to temperatures below CT_max_. However, CT_max_ is a popular measurement because it provides a repeatable [16–18], objective, high-throughput physiological trait that correlates with other thermal traits, including biogeography [19], optimal temperatures for growth [20], and preferred temperatures [20]. Furthermore, to encompass the range of rates of warming used in previous studies [3,9,11], include more ecologically relevant rates of warming, and to investigate if warming rates interact with an oxygen limitation, eight species were also tested using a slower warming rate of 1°C h^−1^. By measuring the individual warming tolerance of 1,451 animals (S2 Table) across 24 experiments and 147 CT_max_ trials, the data presented in this study provide the most comprehensive evaluation to date of the possibility for oxygen supersaturation to improve the resilience of aquatic ectotherms to heatwaves.

## Results

**Fig 2:**
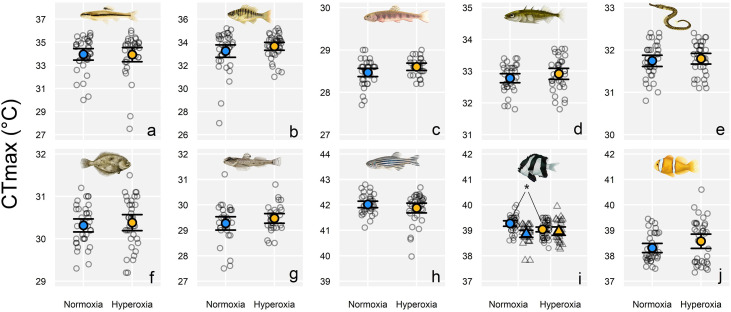
Tolerance to fast warming (0.3°C min^−1^) under normoxia and hyperoxia in 10 tropical and temperate fishes. Shown is the temperature at which loss of motor function occurred (CT_max_) under normoxia (blue; 100% air saturation) and hyperoxia (yellow; *ca*. 150% air saturation). The large symbols show mean values, with individual raw data points scattered behind (error bar = 95% CI). Significant treatment effects (*P* < 0.01) denoted with an * (statistics in S3 Table). Species and sample sizes (*n* = normoxia, hyperoxia) were as follows: **(a)** bluntnose minnow *Pimephalus notatus* (35, 34), **(b)** bluegill *Lepomis macrochirus* (38, 37), **(c)** brook trout *Salvelinus fontinalis* (36, 26), **(d)** three-spined stickleback *Gasterosteus aculeatus* (35, 35), **(e)** lesser pipefish *Syngnathus rostellatus* (36, 35), **(f)** European flounder *Platichthys flesus* (36, 35), **(g)** sand goby *Pomatoschistus minutus* (31, 30), **(h)** zebrafish *Danio rerio* (34, 35), **(i)** humbug damselfish *Dascyllus aruanus* in 2023 (36, 46) (left—circles), and in 2024 (28, 26) (right—triangles), and **(j)** Polynesian anemonefish *Amphiprion maohiensis* (36, 36). Raw data are archived on figshare: https://doi.org/10.6084/m9.figshare.30043432.

**Fig 3:**
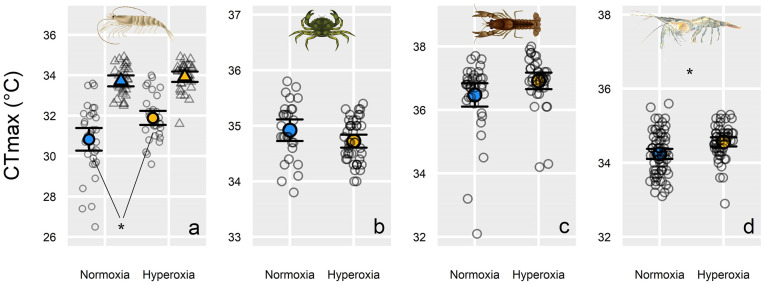
Tolerance to fast warming (0.3°C min^−1^) under normoxia and hyperoxia in four temperate decapod crustaceans. Shown is the temperature at which loss of motor function occurred (CT_max_) under normoxia (blue; 100% air saturation) and hyperoxia (yellow; *ca*. 150% air saturation). The large symbols show mean values, with individual raw data points scattered behind (error bar = 95% CI). Species and sample sizes (*n* = normoxia, hyperoxia) were as follows: **(a)** left: brown shrimp *Crangon crangon* in 2022 (35, 35) (left - circles) and in 2024 (29, 30) (right - triangles), **(b)** green crab *Carcinus maenas* (28, 42), **(c)** rusty crayfish *Faxonius rusticus* (37, 37), and **(d)** Baltic prawn *Palaemon adspersus* (70, 53). Significant treatment effects (*P* < 0.01) denoted with an * (statistics in S3 Table). Raw data are archived on figshare: https://doi.org/10.6084/m9.figshare.30043432.

In the fast-warming experiments (0.3°C min^−1^ warming rate), hyperoxia did not increase warming tolerance (CT_max_) in 12 of 14 species (Figs 2 and 3, and S3 Table). One exception was the brown shrimp *Crangon crangon* in 2022 (Fig 3A), where hyperoxia increased warming tolerance by 1.06°C ± 0.67°C (effect size; mean ± 95% confidence interval; *P* = 0.002). However, in a second set of trials on brown shrimp in 2024, the effect did not occur (*P* = 0.28, Fig 3A). In Baltic prawn, hyperoxia increased CT_max_ by 0.27°C ± 0.18°C (*P* = 0.003; Fig 3D). Hyperoxia decreased CT_max_ by 0.23°C ± 0.14°C in humbug damselfish *Dascyllus aruanus* in our first experiment on the species in 2023 (*P* = 0.002; Fig 2I), but the effect did not occur in a second set of CT_max_ trials conducted in 2024 (*P* = 0.36, Fig 2I). In brook trout *Salvelinus fontinalis,* there was a tendency for an increase (0.16°C ± 0.14°C) in warming tolerance with hyperoxia (*P* = 0.02; Fig 2C), but this did not reach our threshold for statistical significance (α = 0.01; see Materials and methods). Overall, when pooling the fast-warming experiments into a single model (with species-specific random intercepts), there was a negligible effect of hyperoxia (0.18°C ± 0.16°C; *P* = 0.02; Figs 1 and S3).

**Fig 4:**
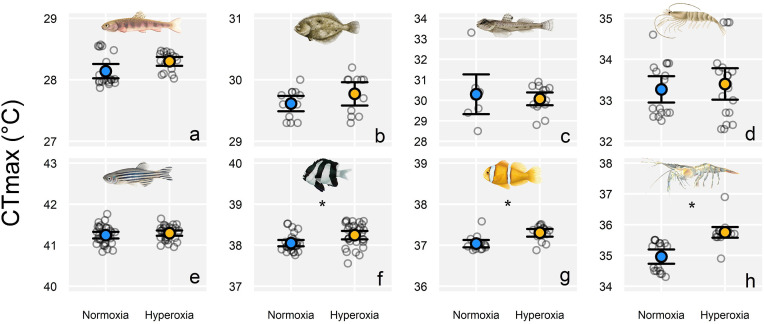
Tolerance to slow warming (1°C h^−1^) under normoxia and hyperoxia in eight temperate and tropical ectotherms. Shown is the CT_max_ under normoxia (blue; 100% air saturation) and hyperoxia (yellow; *ca*. 150% air saturation). The large symbols show mean values, with individual raw data points scattered behind (error bar = 95% CI). Species and sample sizes (*n* = normoxia, hyperoxia) were as follows: **(a)** brook trout *Salvelinus fontinalis* (19, 17), **(b)** European flounder *Platichthys flesus* (13, 11), **(c)** sand goby *Pomatoschistus minutus* (8, 15), **(d)** brown shrimp *Crangon crangon* (16, 19), **(e)** zebrafish *Danio rerio* (31, 29), **(f)** humbug damselfish *Dascyllus aruanus* (28, 30), **(g)** Polynesian anemonefish *Amphiprion maohiensis* (15, 15), and **(h)** Baltic prawn *Palaemon adspersus* (17, 17). Zebrafish and humbug damselfish slow-warming trials involved two replicate CT_max_ trials per treatment; all other species were based on a single slow-warming replicate trial per treatment. Significant treatment effects (*P* < 0.01) denoted with an * (statistics in S3 Table). Raw data are archived on figshare: https://doi.org/10.6084/m9.figshare.30043432.

In the slow-warming experiments (1°C h^−1^ warming rate), which we ran using 8 of 14 species, there was no effect of hyperoxia in 5 of the 8 species. In the Polynesian anemonefish *Amphiprion maohiensis,* there was an increase in CT_max_ of 0.27°C ± 0.14°C (mean ± 95% CI) with hyperoxia (*P* < 0.001; Fig 4G), while a hyperoxia-induced increase in CT_max_ of the humbug damselfish was smaller (0.19°C ± 0.14°C, *P* = 0.007, Fig 4F). In Baltic prawn, the hyperoxia trial had a mean CT_max_ that was 0.79°C ± 0.31°C higher than the corresponding normoxia trial (*P* < 0.001, Fig 4H). Notably, due to their duration, these slow-warming experiments had far fewer replicate animals and trials (typically one replicate trial per treatment) than did our fast-warming experiments, which typically had four replicate trials per treatment (S2 Table). As in the fast-warming experiments, the overall effect across all species in the slow-warming experiments was a tendency for a slight increase of CT_max_ with hyperoxia (0.20°C ± 0.10°C) (*P* < 0.001) (S1 Fig and S3 Table). Across crustaceans (fast and slow warming combined), the mean effect of hyperoxia was clearer (0.39°C ± 0.32°C; *P* = 0.01) than in fishes (not significant: 0.11°C ± 0.10°C; *P* = 0.03; Fig 1 and S3 Table).

Across our experiments (fast and slow warming combined), body mass had a positive effect on warming tolerance in 5 of the 24 experiments, and a negative effect in two experiments (S1 Fig and S3 Table). In most cases, any effect of body mass that did arise was weak (S1 Fig). Importantly, we did not find an interaction between oxygen saturation and body mass in any of the experiments. In general, however, the range in body mass was low in each experiment because our study was not designed to assess the size-dependency of warming tolerance.

## Discussion

The data here provide the most comprehensive assessment to date of the effect of oxygen supersaturation on warming tolerance in aquatic ectotherms. For most of the species and heating rates (i.e., 19 of the 24 experiments), hyperoxia did not increase warming tolerance, resulting in an estimated increase of 0.19°C ± 0.12°C as the overall effect size across the study (Fig 1). In 4 of the 14 species, we did see evidence for small increases in CT_max_ under hyperoxia. The largest effect size was in brown shrimp, which benefited from a *ca.* 1°C mean increase in CT_max_ with hyperoxia during fast warming during our initial experiment on the species in 2022. However, the effect did not occur in a second set of experiments on the species two years later. In Baltic prawn, Polynesian anemonefish, and humbug damselfish, small effects of hyperoxia were detected (0.19–0.79°C increases in warming tolerance; Fig 1). Collectively, our data suggest that the oxygen supersaturation that commonly occurs in shallow, productive aquatic ecosystems is unlikely to provide meaningful survival benefits for most ectotherms during heatwaves.

Unlike our findings, warming tolerance increased substantially in all six species under hyperoxia (140% air saturation) in 2°C h^−1^ warming rate experiments on ectotherms from the Red Sea [3], with the increases ranging from 1.2°C to 3.5°C. One of the species we tested, humbug damselfish *D. aruanus*, was also measured in that study and thus offers a point of direct comparison [3]. In our first experiment with humbug damselfish, we found that hyperoxia caused a small decrease (0.23°C) in warming tolerance [3]. Our second experiment on humbug damselfish, the following year, yielded no effect of hyperoxia in the fast-warming trials. We did see a small hyperoxia-induced improvement in CT_max_ (+0.19°C) in our slow-warming trial for this species, a fraction of the improvement of 1.8°C reported previously [3]. While population differences in thermal tolerance are certainly possible (e.g., due to differences in acclimation history) [21,22], population differences in the effect of hyperoxia on warming tolerance are perhaps less likely, as these would require different physiological mechanisms limiting thermal tolerance [23,24]. The differences in the effects of hyperoxia between our study and that of Giomi and colleagues [3] cannot be explained by differences in warming rate; we used warming rates that encompassed those used previously. One difference was that Giomi and colleagues [3] used median lethal time (LT_50_; temperature at which 50% of animals died) instead of CT_max_, checking on groups of animals (for mortality) every 30 min. While LT_50_ differs from CT_max_, it is generally accepted that death closely follows CT_max_ (i.e., seconds or minutes later, [25]) and therefore LT_50_ and CT_max_ should be broadly comparable. However, in their use of LT_50_, Giomi and colleagues [3] only generated one estimate of warming tolerance for each species and treatment, with no replicate trials (precluding the use of statistics). Modest variations in abiotic environmental factors other than temperature (e.g., salinity, dissolved CO_2_, pH) typically have limited effects on warming tolerance in aquatic organisms, so these seem unlikely to be responsible for stark differences in the effects of hyperoxia across studies [26–30]. While we cannot rule out unknown sources of biotic or abiotic variation as explanations of differences between our results and those of Giomi and colleagues [3], ultimately, we are confident in our estimates of the effects of hyperoxia given the statistical power and replication in our study.

Most studies that have assessed the effect of hyperoxia on warming tolerance across tropical, temperate, and Antarctic fish species have either found no effect or a relatively small positive effect (typically <1°C, reviewed by [9]). However, of the previous studies that have found small increases in CT_max_ in hyperoxia, many involved small sample sizes and a single warming tolerance trial per treatment. For tests of warming tolerance like CT_max_, it is valuable to conduct multiple replicate trials per treatment to obtain accurate estimates of treatment effects. Our results show that even with multiple replicate trials (each with several animals), small, context-specific treatment differences may not occur when an experiment is repeated, as occurred here with humbug damselfish and brown shrimp. We ran four replicate CT_max_ trials in most cases for the fast-warming experiments (sample sizes in S2 Table), providing a glimpse into inter-trial variability within treatments (S3 Fig and S4 Table). Even with the same experimenter scoring CT_max_ on the same species, we found that there was often a range of 0.5°C or more in mean CT_max_ among replicate trials (7–10 animals per trial), with larger inter-trial differences in mean CT_max_ of 2.5–3°C in 2 of 13 species (brown shrimp and bluntnose minnow; S3 Fig and S4 Table). Thus, a treatment effect for CT_max_ (or LT_50_) should be interpreted with caution if based on a single trial per treatment (or low sample sizes generally), especially if the effect size is small (e.g., 0.5°C or less), as has been the case in several previous studies on the effects of hyperoxia on warming tolerance and in some of the slow-warming experiments we conducted here.

Given the predictions of the oxygen-limitation hypothesis [7], directly removing any limit to oxygen supply via supersaturation can be an elegant way to experimentally assess the role of oxygen in warming tolerance [2]. Indeed, of the 18 studies that have measured the partial pressure of oxygen in arterial blood (PaO_2_) in fish acclimated for hours or days to hyperoxia, nearly all have found substantial increases in PaO_2_ [5]. Of those studies that used hyperoxia within the range of our study (*ca*. 125%–175% air saturation), PaO_2_ increased by a factor of *ca*. 1.5–2 in fish [5]. In turn, environmental hyperoxia can enable fish to increase their uptake of oxygen (i.e., maximum aerobic metabolic rate) and aerobic scope (i.e., the difference between standard and maximum aerobic metabolic rates) [31,32]. For example, Skeeles and colleagues [32] found a 74%–95% increase in aerobic scope following acute (~4 h) exposure to hyperoxia (150% air saturation), while Brijs and colleagues [31] also reported close to a doubling of aerobic scope after 14 h of exposure to 200% air saturation. Based on these previous experiments in other species, the fish in our study likely had higher oxygen availability (or, at least, higher O_2_ in circulation) when tested in hyperoxia versus normoxia, yet warming tolerance was unaffected in most cases. Nevertheless, more data across a wider array of species on how standard and maximum rates of oxygen uptake are affected by hyperoxia, especially at supraoptimal temperatures, would be useful to provide context for any effects of hyperoxia on CT_max_.

Ultimately, our data suggest that the presence of oxygen supersaturation during heatwaves in temperate and tropical aquatic habitats is unlikely to improve the survival of most resident ectotherms. Oxygen is crucial to life and can affect the thermal performance and tolerance of water breathers under some contexts [2,5,10,33], especially under moderate or severe hypoxia [10,33,34]. However, with the new dataset presented here, we conclude that incorporating a “protective” effect of oxygen supersaturation into mechanistic species distribution models and climate risk assessments should only occur in cases where robust, well-replicated, species-specific evidence supports an effect of hyperoxia [35–37]. Otherwise, assuming a protective effect of hyperoxia could risk overestimating the resilience of aquatic animals to climate warming.

## Materials and methods

### Study sites, species, and holding conditions

We used 14 species for this study (Fig 1), 12 of which were wild animals we captured in the field and brought into the laboratory for experimentation. In each set of experiments described below, once in captivity, the animals were exposed to photoperiods that approximated natural photoperiods for that location and time of year. The first series of experiments, on temperate marine species, took place in 2022 at Kristineberg Marine Station (animal ethics permit #Dnr 5.8.18-8955/2022 issued to Jutfelt from the Ethical Committee for Animal Research in Gothenburg), Sweden, by the Gullmars Fjord, Skagerrak Sea (58.24965 N, 11.44585 E). All collection and experiments at Kristineberg Marine Station (Sweden) were conducted in accordance with the EU legislation on animal welfare (Directive 2010/63/EU and Regulation (EU) 2019/1010) and national laws; Swedish Animal Welfare Act (2018:1192). We collected four marine fishes (sand goby *Pomatoschistus minutus*, three-spined stickleback *Gasterosteus aculeatus*, lesser pipefish *Syngnathus rostellatus*, European flounder *Platichthys flesus*) and two marine decapod crustaceans (brown shrimp *C. crangon*, green crab *Carcinus maenas*) by beach seine (1 × 8 m, 3 mm mesh) in shallow (<1 m) coastal environments that periodically exhibit hyperoxia (S2 Fig). Animals were acclimated to the laboratory for at least 24 h before being used in CT_max_ trials, in tanks supplied with constant flow-through of seawater supplied from the fjord (in normoxia, ambient temperatures, mean ± S.D. 16.26°C ± 0.66°C for sand shrimp and green crab, 17.54°C ± 0.97°C for the other species). Fish and decapods were fed once daily to apparent satiation with freshly thawed mysid (Akvarie Teknik) and *Pandalus borealis* shrimp and newly hatched artemia but were fasted for 24 h prior to use in CT_max_ trials. Salinity averaged *ca*. 27–28 ppt in the laboratory during these experiments (in holding tanks and therefore also in the experimental water used in CT_max_ arenas).

The second set of experiments, on temperate freshwater species, took place in 2022 in the laboratory at Trent University (hereafter, Trent U), Canada (44.359499 N, 78.289008 W; animal ethics permit #28105 issued to Raby by the Trent U Animal Care Committee) with four freshwater species. Two species (bluegill *Lepomis macrochirus* [young-of-year] and bluntnose minnow *Pimephalus notatus* [juveniles and adults]), were collected within 2 km of Trent U from the Otonabee River using a beach seine (15 × 1.5 m, 3 mm mesh; License to Collect Fish for Scientific Purposes #1102029 issued to Raby by the Ontario Ministry of Natural Resources). The same beach seine was used to collect rusty crayfish, *Faxonius rusticus* (juveniles and adults), from a pond on the Trent U campus. The fourth species used for experiments at Trent U was brook trout *S. fontinalis* (juveniles), which were provided by the Codrington Fisheries Research Facility (Ontario Ministry of Natural Resources, 44.14760 N, 77.80190 W) after being incubated and raised (to ~6 months post-hatching) from the gametes of spawning fish caught in Salt Creek, ON (44.149889 N, 77.940750 W), in the autumn of 2021. A second group of brook trout (2 months post-hatch) were later brought from the same hatchery to Trent U for slow warming (1°C h^−1^) CT_max_ trials in spring of 2023. Each of these species were fed daily with bloodworms and/or commercial pellets but left unfed on the day they were tested (typically a 20–24 h fasting period), with tests generally commencing 1–2 days after fish arrived in the laboratory. At Trent U, animals were held in tanks which were continuously refreshed with water from the Otonabee River that was sand-filtered and disinfected with an ozonation system. Each tank was also aerated with an air stone and further filtered with an aquarium canister filter. The tanks were thermostatically controlled to maintain a stable temperature matching (within *ca*. 2°C) the temperature at which fish were collected (rusty crayfish mean ± S.D. = 18.21°C ± 0.69°C; bluntnose minnow = 21.24°C ± 0.24°C; bluegill = 18.31°C ± 0.51°C; brook trout = 8.25°C ± 0.36°C).

The third set of experiments, on a tropical marine species, took place at CRIOBE research station in Moorea, French Polynesia, in 2023 (Ethical approval was granted by the CNRS Animal Experimentation permit numbers R-13-CNRS-F1-16 and 006725, and ANZCCART ComPass Animal Welfare Training certificate). Humbug damselfish *D. aruanus* (juveniles and adults) were collected while snorkeling in shallow coral reefs at Papetō’ai, northern Moorea. The fish were then quickly transported to holding tanks (100 L), where they were kept for one week prior to the experiments. Both collection site temperatures and holding tank temperatures were 28°C–29°C. The tanks had continuous flow through seawater, and fish were fed dry feed daily, except in the last 24 h prior to the experiments.

The fourth set of experiments used zebrafish *Danio rerio*, a tropical freshwater species, in the laboratory at the Norwegian University of Science and Technology (NTNU) (63.41890 N, 10.40265 W; animal ethics permit #29878 issued to Jutfelt by the Norwegian Food Safety Authority) in 2023. The zebrafish were 8th-generation offspring from wild fish collected in Northwest Bengal, India, in 2016 [38]. The fish had been acclimated to a constant temperature of 28°C for a year prior to the CT_max_ trials. Each holding tank (60 × 35 × 30 cm) was aerated using an air stone and contained a sponge filter and had a low rate of continuous water replacement. All individuals were fed twice every day with commercial flakes (TetraPRO Energy Multi-Crisp) but were fasted on the day of CT_max_ trials.

The fifth set of experiments, on two tropical marine species, took place again at CRIOBE research station in Moorea, but in 2024 (Ethical approval was granted from the CNRS Animal Experimentation permit numbers R-13-CNRS-F1-16 and 006725, and ANZCCART ComPass Animal Welfare Training certificate). Humbug damselfish (juveniles and adults) were collected while scuba diving in shallow coral reefs (*ca*. 2 m depth) at different locations on the North coast of Moorea (Animal collection was granted from The Ministere de l’Agriculture et des Ressources Marines, en charge de l’Alimentation et de la Recherche, et de la Cause animale [MPR] of French Polynesia permit numbers 8286-MPR/DIREN and 7445/MPR/DRM, within the framework of the BLEACHALAN, Raising Nemo, and AUFRANDE projects). Upon collection, fish were quickly transported to holding tanks (100 L) where they were allowed to acclimate for a minimum of one week prior to experiments. Polynesian anemonefish, *A. maohiensis* (juveniles; [39]), were obtained from Coopérative des Aquaculteurs de Polynésie Française (C.A.P.F.) at Tahiti, and transported to CRIOBE research station in Moorea, where they arrived in March 2024 and were quickly transferred to their holding tanks (100 L; acclimatized for a minimum of one week prior to experiments). Holding tank temperatures ranged between 29°C and 31°C. The tanks had continuous flow through seawater, and fish were fed live *Artemia* spp., except in the last 24 h prior to the experiments. Animal care in French Polynesia adhered to the National Charter on the Ethics of Animal Experimentation developed by the Comité National de Réflexion Ethique sur l’Expérimentation Animale (French national committee for consideration of ethics in animal experimentation; CNREEA).

The sixth and final set of experiments, on temperate marine species, took place at Kristineberg Marine Station (animal ethics permit #Dnr 5.8.18-07417/2024 issued to Jutfelt from the Ethical Committee for Animal Research in Gothenburg) in 2024. Two marine decapod crustaceans (brown shrimp and Baltic prawn *Palaemon adspersus*) were collected via beach seine in shallow coastal environments. Animal acclimation and holding were similar to our first set of experiments at the same location in 2022. The mean acclimation temperatures ± S.D. in holding tanks were 18.3°C ± 0.63°C for brown shrimp and 18.42°C ± 0.54°C for Baltic prawn (mean salinity ranging from 25 to 28 ppt). Decapods were fed once daily with thawed *P. borealis* shrimp and were fasted the day of CT_max_ trials. The animals for these experiments were held in the laboratory for at least 24 h (up to 5 days) prior to use in CT_max_ trials.

### Measurement of critical thermal maximum (CT_max_)

For all 14 species, we followed a standardized method for CT_max_, with a warming rate of 0.3°C min^−1^ [18]. In 8 of the 14 species (sand goby, European flounder, brook trout, zebrafish, Polynesian anemonefish, humbug damselfish, brown shrimp, Baltic prawn), we conducted additional CT_max_ trials with a warming rate of 1°C h^−1^. Animals were placed into the arena to habituate for 30 min before warming began (at either normoxia [100% air saturation] or hyperoxia [150%], matching their holding acclimation temperature), except for the 2024 experiments with Baltic prawn and brown shrimp, which were given 10 min of arena habituation time. Heaters were then switched on, achieving a warming rate of 0.3°C min^−1^ (or 1°C h^−1^), with identical water volume and heating power used for all trials for a given species, such that warming rates were consistent among replicate trials (photos of CT_max_ arenas we used in S4 Fig). We conducted 3–5 CT_max_ trials per species and oxygen treatment (normoxia and hyperoxia), with **n* *= 7–10 animals per trial to achieve sample sizes of *n* ~ 35 per oxygen treatment and species in most cases, and one or two trials per treatment (and species) for the slow-warming experiments (sample sizes in S2 Table). For the normoxia treatment, aeration with an air stone ensured the arena stayed close to 100% air saturation (typically 95%–105%). For the hyperoxia treatment, a similar air stone connected to a cylinder of compressed O_2_ was used to bubble O_2_ into the arena until dissolved oxygen (DO) reached ~150% air saturation. DO was then monitored carefully, with regular adjustments to ensure DO remained within ~5% of 150%. To monitor and record DO and temperature for experiments at Kristineberg (2022) and Trent U, we used a YSI ProSolo ODO Optical Dissolved Oxygen Meter (https://www.ysi.com/prosolo-odo), with the meter set to log DO and temperature at 30 s intervals. For all other experiments, we used a PyroScience Firesting-O_2_ Optical Oxygen and Temperature Meter (https://www.pyroscience.com/) (recording rate of 1 Hz). For most of the trials at Trent U and Kristineberg (2022), we also logged temperature in the CT_max_ arena using an RBR ProSolo Temperature logger (https://rbr-global.com/) set to log temperature every 10 s. Raw data for temperature and oxygen from our CT_max_ trials are visualized in a supplementary file available in the figshare repository for this paper: https://doi.org/10.6084/m9.figshare.30043432.

CT_max_ was quantified as the temperature at which each animal lost equilibrium (i.e., righting reflex). Because we studied a diversity of organisms, these endpoints differed slightly in the way they were assessed among species. For most fishes, loss of equilibrium was defined as the point where they could not maintain a stable upright position for three continuous seconds [40]. For the decapod crustaceans, CT_max_ was typically preceded (immediately) by bursting up off the bottom of the arena, then drifting back to the bottom with negative equilibrium. However, we also used a small dip net or plastic probe to frequently turn the invertebrates upside-down to check whether they maintained their righting reflex. For any given experiment, the same person scored CT_max_ for all animals for both treatments, and that person was always blinded to temperature. That is, a second person monitored temperature and oxygen, and recorded the temperature at which each animal was removed from the arena (i.e., its CT_max_ value). Animals were transferred into individual recovery containers following CT_max_ and given at least 10 min to recover (to confirm they regained equilibrium and normal ventilation). Each animal was then euthanized with a lethal overdose of tricaine methanesulfonate (MS-222, Pharmaq) or clove oil (C8392, Sigma Aldrich) before being weighed and measured, with the exception of the humbug damselfish in Moorea and decapods at Kristineberg Marine Station in 2024, which were released after being weighed, measured, and recovered overnight.

### Statistics

The effect of oxygen treatment on CT_max_ was modeled separately for each species using linear models with body mass (log-transformed) as a covariate and an interaction between mass and oxygen treatment (normoxia, hyperoxia). The interaction was removed if it was not significant (α = 0.05). Likewise, if mass had no effect on CT_max_ (α = 0.05), it was removed from the model. We tested for the effect of hyperoxia on CT_max_ in 14 species for the fast-warming trials (0.3°C min^−1^, including two separate models for two sets of humbug damselfish experiments), and separately for slow-warming trials (8 of 14 species, 1°C h^−1^), for 24 models in total (linear models). In addition, to generate an overall effect size estimate (i.e., aggregating all 1,451 data points), we ran a linear mixed effects (using the “lme” function from the “nlme” package in R [41]) model using oxygen treatment as a fixed effect and experiment (i.e., each species × warming rate combination) as a random effect (random intercept and random slope, i.e., “random = ~1 + oxygen treatment | experiment ID” allowing slopes and intercepts to vary for the 24 experiments). We used the same mixed effects model approach to generate effect-size estimates for fish, crustaceans, slow warming experiments, and fast warming experiments as larger groups (i.e., in each case, experiment ID was used as a random effect, as above). In most cases with these group models, a random term using random slopes and intercepts provided better fit than using only random intercepts (based on ∆AIC and log-likelihood tests). There were two exceptions: for the fish model and for the slow warming model, adding a random slope did not improve model fit (so only random intercept models were used). Given that we conducted 29 separate statistical tests (24 experiments + 5 aggregate tests of different subgroups) of the null hypothesis that hyperoxia does not affect warming tolerance (CT_max_), we wished to guard against type I errors via an adjustment to our significance threshold (α). However, Bonferroni corrections (dividing 0.05 by the number of tests, in our case 0.05/29 = 0.002) can be overly conservative [42], resulting in a high risk of type II errors. Thus, to strike a balance between avoiding type I and type II errors, we set α to an intermediate value of 0.01. However, recognizing that *P* values can be viewed at as a continuum of the strength of evidence (rather than a binary test; [43], and that null hypothesis statistical testing has been criticized [44], we place emphasis on effect sizes in our interpretations. Model assumptions were assessed by visual inspection of residuals. Analyses were conducted using R (v.4.4.1 [45]) with RStudio (v.2024.09.0 [46]).

## Supporting information

S1 Table The range of oxygen supersaturation that occurs in the ecosystems relevant to the species included in our study. Hyperoxia (dissolved oxygen partial pressures >100% air saturation) in the wild is evident from several studies from the “early 90s to early 2020s”. In general, the phenomenon occurs when primary producers release oxygen from photosynthesis into water, and warming simultaneously decreases the water’s oxygen solubility (Giomi and colleagues 2019). Aquatic ecosystems with a high proportion of primary producers relative to respiring animal biomass, easy access of sunlight due to shallow depth, and limited water exchange can become saturated with oxygen, and a relative increase in temperature will therefore supersaturate the water, even at temperatures that might not be perceived as “warm”. The time of the day when the water heats up the fastest also varies depending on the ecosystem. For example, midday is reported in the tropics, where a zenithal sun position provides the strongest energy input (Giomi and colleagues 2019). In contrast, late afternoon can be the warmest time in the northern hemisphere, where a colder climate and lower angle of the sun slows down heat transfer and creates a lag. Heating rate is further affected by how isolated the water is and can thus be influenced by tidal cycles in closed-off bays, lagoons, tidal marshes, and rock pools.
(DOCX)

S2 Table Sample sizes and body mass for each of the 24 sets of CT_max_ experiments for this study. Fast warming = 0.3°C min^−1^, slow warming = 1°C h^−1^.
(DOCX)

S3 Table Model estimates for normoxia (intercept) and for the effects of hyperoxia for each of the 24 experiments modeled with separate linear models for each species. The mass covariate (log transformed) was removed if not significant (*P* > 0.05) in the final model, but we give the mass coefficient estimate and *P* values from the full model in those cases where it was not significant. The bottom five models are based on linear mixed effects models with random intercepts and slopes, except for the “fish” model and the “slow warming” model, which were fit better using random intercepts only (based on comparison of AIC values and log-likelihood tests).
(DOCX)

S4 Table Statistics describing variation in CT_max_ among fast-warming (0.3°C min^−1^) replicate trials within a species and treatment (3–5 replicate trials per group, *ca*. 7–10 animals per replicate, see S2 Table for sample sizes). The *F* and *P* values are from ANOVAs testing for differences among replicate CT_max_ trials. The CT_max_ mean range refers to the difference between the highest and lowest mean within-trial CT_max_ values. The data are visualized in S3 Fig.
(DOCX)

S1 Fig Temperature at which loss of motor function occurred (CT_max_) in 24 experiments including 14 species of aquatic ectotherms, as a function of body mass (log_10_-transformed, as in our statistics). Animals from the normoxia treatment are shown in blue circles, hyperoxia in yellow diamonds. Linear relationships are shown where they were statistically significant (*P* < 0.01, see S3 Table). The 16 top panels are from the fast-warming trials (0.3°C min^−1^), the bottom eight panels shaded in blue are the slow-warming (1°C h^−1^) trials. The species are as follows: (i) bluntnose minnow *Pimephalus notatus*, (ii) bluegill *Lepomis macrochirus*, (iii) brook trout *Salvelinus fontinalis*, (iv) three-spined stickleback *Gasterosteus aculeatus*, (v) lesser pipefish *Syngnathus rostellatus* (vi), European flounder *Platichthys flesus* (vii), sand goby *Pomatoschistus minutus*, (viii) zebrafish *Danio rerio*, (ix) humbug damselfish *Dascyllus aruanus* experiment 1 (2023), (x) humbug damselfish experiment 2 (2024), (xi) Polynesian anenomefish *Amphiprion maohiensis*, (xii) green crab *Carcinus maenas*, (xiii) rusty crayfish *Faxonius rusticus*, (xiv) brown shrimp *Crangon crangon* experiment 1 (2022), (xv) brown shrimp *C. crangon* experiment 2 (2024), (xvi) Baltic prawn *Palaemon adspersus*, (xvii) sand goby, (xviii) European flounder, (xix) brook trout, (xx) zebrafish, (xxi) humbug damselfish, (xxii) Polynesian anenomefish, (xxiii) Baltic prawn, (xxiv) brown shrimp.
(DOCX)

S2 Fig Measurements of supersaturation in the area where the following species were collected for the study’s experiments: lesser pipefish *Syngnathus rostellatus*, three-spine stickleback *Gasterosteus aculeatus*, sand goby *Pomatoschistus minutus*, European flounder *Platichthys flesus*, green crab *Carcinus maenas,* and brown shrimp *Crangon crangon.* In short, 9−10 seagrass *Zostera marina* meadows, where all the above-listed species were found, within 10 km of Kristineberg Marine Station (58.24965 N, 11.44585 E), were sampled using a handheld oximeter at 1 m depth for temperature, salinity, and dissolved oxygen in June, September, and October 2022. The oxygen saturation point was then calculated using the o2.at.sat function in the LakeMetabolizer (Winslow and colleagues, 2016, https://doi.org/10.1080/IW-6.4.883) package with the “garcia-benson” model applied to the data. From this, the oxygen saturation level of each site and date was calculated as %O_2_ = O_2_/O_2_′ × 100, where O_2_ was the dissolved oxygen in the sample in mg L^−1^ and O_2_′ was the oxygen solubility for each measurement of salinity and temperature. Blue circles show the calculated oxygen saturation. Green triangles show the corresponding temperature (shown on the right y-axis) measured at each site and date. Lines show the average value for all measurement points and months.
(DOCX)

S3 Fig CT_max_ data for fast-warming (0.3°C min^−1^) plotted separately by replicate trials, with individual data points shown and mean (yellow = hyperoxia, blue = normoxia) and 95% confidence intervals plotted for each group. Sample sizes are given in S2 Table. **a**: bluntnose minnow, **b**: bluegill, **c**: brook trout, **d**: zebrafish, **e**: threespine stickleback, **f**: lesser pipefish, **g**: sand goby, **h**: European flounder, **i**: humbug damselfish experiment 1 (2023), **j**: humbug damselfish experiment 2 (2024), **k**: Polynesian anemonefish, **l**: brown shrimp experiment 1 (2022), **m**: brown shrimp experiment 2 (2024), **n**: green crab, **o**: rusty crayfish, **p**: Baltic prawn. See S1 Fig caption for scientific names.
(DOCX)

S4 Fig Overhead photos of CT_max_ arenas we used. **a**: The arena we used for stickleback, zebrafish, lesser pipefish, sand goby, green crab, brown shrimp, and European flounder with a total water volume of 12 L. **b**: The arena we used for humbug damselfish and Polynesian anemonefish in 2024 with a water volume 8 L for fast-warming, 18 L for slow-warming; a similar arena was used in 2023 (humbug damselfish). **c**: the arena we used for brook trout, bluntnose minnow, rusty crayfish, and bluegill, with a water volume of 26 L. **d**: the arena (left = arena where the fish were confined, right = sump containing heaters, pumps, and air stones) that we used for the slow-warming sand goby and flatfish trials with a total water volume of 35 L.
(DOCX)

## Abbreviations

CT_max_: critical thermal maximum
DO: dissolved oxygen
