# Correction: Oxygen supersaturation has negligible effects on warming tolerance across diverse aquatic ectotherms

**DOI:** 10.1371/journal.pbio.3003626

**Published:** 2026-01-28

**Authors:** Graham D. Raby, Jeremy De Bonville, Leroy Reynolds, Zoe Storm, Zara-Louise Cowan, Moa Metz, Anna H. Andreassen, Leon Pfeufer, Emily R. Lechner, Erin M. C. Stewart, Robine H. J. Leeuwis, Rasmus Ern, Lorena Silva-Garay, Michael R. Skeeles, Dominique G. Roche, Rachael Morgan, Leon Green, Ben Speers-Roesch, Suzanne C. Mills, Timothy D. Clark, Fredrik Jutfelt

The Funding statement for this article is incorrect. The correct Funding statement is as follows: This study was funded by a European Research Council Consolidator grant (CLIMEVOLVE; to FJ) and by the Natural Sciences and Engineering Research Council of Canada (Discovery Grants to GDR and to B-SR). TDC was supported by an Australian Research Council Future Fellowship (FT180100154) funded by the Australian Government. RE received funding from the European Union’s Horizon 2020 research and innovation programme under the Marie Skłodowska-Curie grant agreement No 893895. This project also benefitted from co-funding between the EU program for research and innovation Horizon Europe and Marie Skłodowska-Curie no. 101081465 (AUFRANDE) to SCM and Pacific Funds “BLEACHALAN” and Recherche et Innovation Partenariat Public Privé RIP4 “Raising Nemo” to SCM. The funders had no role in study design, data collection and analysis, decision to publish, or preparation of the manuscript.
