# Supplementary material for: Oxygen supersaturation has negligible effects on warming tolerance across diverse aquatic ectotherms
Source: PLoS Biol. 2025 Nov 4;23(11):e3003413. doi: 10.1371/journal.pbio.3003413 (PMC12585006; doi:10.1371/journal.pbio.3003413)
Supplement: S1 Table — Hyperoxia (dissolved oxygen partial pressures >100% air saturation) in the wild is evident from several studies from the “early 90s to early 2020s”. In general, the phenomenon occurs when primary producers release oxygen from photosynthesis into water, and warming simultaneously decreases the water’s oxygen solubility (Giomi and colleagues 2019). Aquatic ecosystems with a high proportion of primary producers relative to respiring animal biomass, easy access of sunlight due to shallow depth, and limited water exchange can become saturated with oxygen, and a relative increase in temperature will therefore supersaturate the water, even at temperatures that might not be perceived as “warm”. The time of the day when the water heats up the fastest also varies depending on the ecosystem. For example, midday is reported in the tropics, where a zenithal sun position provides the strongest energy input (Giomi and colleagues 2019). In contrast, late afternoon can be the warmest time in the northern hemisphere, where a colder climate and lower angle of the sun slows down heat transfer and creates a lag. Heating rate is further affected by how isolated the water is and can thus be influenced by tidal cycles in closed-off bays, lagoons, tidal marshes, and rock pools. (DOCX) [file pbio.3003413.s001.docx]

**Supplementary Information** **for**
*Oxygen supersaturation has negligible effects on warming tolerance across diverse aquatic ectotherms*

**S1 Table.** The range of oxygen supersaturation that occurs in the ecosystems relevant to the species included in our study. Hyperoxia (dissolved oxygen partial pressures >100% air saturation) in the wild is evident from several studies from the early ‘90s to early 2020’s. In general, the phenomenon occurs when primary producers release oxygen from photosynthesis into water and warming simultaneously decreases the water’s oxygen solubility (Giomi et al. 2019). Aquatic ecosystems with a high proportion of primary producers relative to respiring animal biomass, easy access of sunlight due to shallow depth and limited water exchange can become saturated with oxygen, and a relative increase in temperature will therefore supersaturate the water, even at temperatures that might not be perceived as “warm”. The time of the day when the water heats up the fastest also varies depending on the ecosystem. For example, midday is reported in the tropics, where a zenithal sun position provides the strongest energy input (Giomi et al. 2019). In contrast, late afternoon can be the warmest time in the northern hemisphere, where a colder climate and lower angle of the sun slows down heat transfer and creates a lag. Heating rate is further affected by how isolated the water is and can thus be influenced by tidal cycles in closed-off bays, lagoons, tidal marshes and rock pools.

| **Habitat type and location** | **[DO] (% air saturation)** | **Temp  (°C)** | **Salinity (ppt)** | **Depth (m)** | **Relevant species** | **Source** |
| --- | --- | --- | --- | --- | --- | --- |
| Near shore pelagic, Southern Baltic Sea | 107-132 | 12-17 | 7-12* | <1 | Lesser pipefish, Three-spine stickleback, Sand goby, European flounder, Green crab, Brown shrimp | Marks (2008).  <https://doi.org/10.2166/nh.2008.021> |
| Pelagic, Skagerrak Baltic Sea | 102-115.7 | 12-17 | 7-31 | <15 | Three-spine stickleback, Sand goby, European flounder, Green crab, Brown shrimp | Stigebrandt (1991).  <https://doi.org/10.4319/lo.1991.36.3.0444> |
| Experimental shallow soft bottom community, Baltic Sea | 134 | 18 | 7 | NA | Sand goby, European flounder, Green crab, Brown shrimp | Gorska et al. (2018).  <https://doi.org/10.1016/j.jmarsys.2018.01.001> |
| Pelagic, Baltic Sea | 133-152 | NA | 5-8.6 | <15 | Three-spine stickleback | Rahm et al. (1995).  <https://doi.org/10.1007/BF00552572> |
| Pelagic, North Sea | 124-188 | 15-18* | 33-35* | <4 | Larvae of Sand goby, European flounder, Green crab, Brown shrimp | Riebesell (1992).  <https://doi.org/10.4319/lo.1992.37.1.0063> |
| Surface waters in tidal channels, Wadden Sea | 100-148 | 12-15 | 19-28 | <19 | Lesser pipefish, Three-spine stickleback, Sand goby, European flounder, Green crab, Brown shrimp | Hoppema (1991).  <https://doi.org/10.1016/0272-7714(91)90036-B> |
| Large river, Grand River watershed, Ontario, Canada | 150-180 | 20 | 0* | 0.5 - 1 | Brook trout, Bluegill sunfish, Blunthead minnow, rusty crayfish | Rosamond et al. (2011).  <https://doi.org/10.2134/jeq2010.0009> |
| Meltwater influenced lake, Kootenay Lake, British Columbia, Canada | 121-140 | NA | 0* | 2 | Brook trout, Bluegill sunfish | Northcote et al. (2005).  [doi.org/10.1080/07438140509354434](http://doi.org/10.1080/07438140509354434) |
| Mangrove forest, Red Sea, Saudi Arabia | 100-250 | 28-42 | 42 | NA | Humbug damselfish | Giomi et al. (2019).  [doi.org/10.1126/sciadv.aax1814](http://doi.org/10.1126/sciadv.aax1814) |
| Large river, Kanhan River, Vidharba, India | 151 | 28 | 0* | NA | Zebrafish | Central Water Commission (2019).  <https://cwc.gov.in/sites/default/files/effect-time-and-temperature-do-levels-river-water-2019.pdf> |

*not specified or recorded in the study, but retrieved from other regional environmental data.
