# Supplementary material for: Oxygen supersaturation has negligible effects on warming tolerance across diverse aquatic ectotherms
Source: PLoS Biol. 2025 Nov 4;23(11):e3003413. doi: 10.1371/journal.pbio.3003413 (PMC12585006; doi:10.1371/journal.pbio.3003413)
Supplement: S2 Table — Fast warming = 0.3°C min−1, slow warming = 1°C h−1. (DOCX) [file pbio.3003413.s002.docx]

**Supplementary Information** **for**
*Oxygen supersaturation has negligible effects on warming tolerance across diverse aquatic ectotherms*

**S1 Table.** Sample sizes and body mass for each of the 24 sets of CT_max_ experiments for this study. Fast warming = 0.3°C min^-1^, slow warming = 1°C h^-1^.

| **Species** | **Treatment** | | | | **Warming rate** | | **Body mass, g  (mean, range)** | **Trials (*n*)** | **Animals (total *n* across trials)** |
| --- | --- | --- | --- | --- | --- | --- | --- | --- | --- |
| bluntnose minnow, *Pimephalus notatus* | hyperoxia | | | | fast | | 1.17 (0.22 – 6.96) | 4 | 34 |
|  | normoxia | | | | fast | | 1.21 (0.40 – 8.29) | 4 | 35 |
| brook trout, *Salvelinus fontinalis* | hyperoxia | | | | fast | | 5.39 (2.82 – 10.80) | 3 | 26 |
|  | normoxia | | | | fast | | 6.00 (3.55 – 12.54) | 4 | 36 |
| rusty crayfish, *Faxonius rusticus* | hyperoxia | | | | fast | | 4.86 (0.75 – 29.75) | 4 | 37 |
|  | normoxia | | | | fast | | 3.27 (0.25 – 9.79) | 4 | 37 |
| bluegill sunfish, *Lepomis macrochirus* | hyperoxia | | | | fast | | 0.74 (0.25 – 1.74) | 4 | 37 |
|  | normoxia | | | | fast | | 0.71 (0.16 – 1.49) | 4 | 38 |
| European flounder, *Platichthys flesus* | hyperoxia | | | | fast | | 0.58 (0.28 – 1.78) | 4 | 36 |
|  | normoxia | | | | fast | | 0.61 (0.11 – 1.72) | 4 | 36 |
| green crab, *Carcinus maenas* | hyperoxia | | | | fast | | 1.57 (0.31 – 3.65) | 4 | 42 |
|  | normoxia | | | | fast | | 1.50 (0.31 – 3.38) | 3 | 28 |
| humbug damselfish, *Dascyllus aruanus* | hyperoxia | | | | fast | | 1.34 (0.13 – 4.12) | 5 | 46 |
|  | normoxia | | | | fast | | 1.50 (0.08 – 5.05) | 4 | 36 |
| humbug damselfish experiment 2 | hyperoxia | | | | fast | | 1.23 (0.03 – 6.25) | 3 | 26 |
|  | normoxia | | | | fast | | 0.82 (0.03 – 3.56) | 3 | 28 |
| Polynesian anemonefish, *Amphiprion maohiensis* | hyperoxia | | | | fast | | 1.15 (0.60 – 1.86) | 4 | 36 |
|  | normoxia | | | | fast | | 1.19 (0.58 – 2.24) | 4 | 36 |
| lesser pipefish, *Syngnathus*  *rostellatus* | hyperoxia | | | | fast | | 0.65 (0.24 – 1.19) | 4 | 35 |
|  | normoxia | | | | fast | | 0.70 (0.32 – 1.27) | 4 | 36 |
| sand goby, *Pomatoschistus minutus* | hyperoxia | | | | fast | | 1.82 (0.81 – 3.12) | 4 | 30 |
|  | normoxia | | | | fast | | 1.60 (0.65 – 2.68) | 4 | 31 |
| brown shrimp, *Crangon crangon* | hyperoxia | | | | fast | | 0.70 (0.11 – 1.52) | 4 | 35 |
|  | normoxia | | | | fast | | 0.76 (0.07 – 1.90) | 4 | 35 |
| brown shrimp experiment 2 | hyperoxia | | | fast | | | 0.49 (0.27 – 0.87) | 3 | 30 |
|  | normoxia | | | fast | | | 0.56 (0.19 – 1.47) | 3 | 29 |
| Baltic prawn, *Palaemon adspersus* | | hyperoxia | | fast | | 1.24 (0.33 – 3.24) | | 5 | 53 |
|  |  | normoxia | | fast | | 1.42 (0.37 – 3.87) | | 7 | 70 |
| three-spined stickleback, *Gasterosteus aculeatus* | hyperoxia | | | | fast | | 1.42 (0.86 – 2.39) | 4 | 35 |
|  | normoxia | | | | fast | | 1.81 (0.92 – 2.85) | 4 | 35 |
| zebrafish, *Danio rerio* | hyperoxia | | | | fast | | 0.25 (0.18 – 0.38) | 5 | 35 |
|  | normoxia | | | | fast | | 0.25 (0.14 – 0.42) | 5 | 34 |
| brook trout, *Salvelinus fontinalis* | hyperoxia | | | | slow | | 0.60 (0.46 – 0.77) | 1 | 17 |
|  | normoxia | | | | slow | | 0.57 (0.40 – 0.85) | 1 | 19 |
| European flounder, *Platichthys flesus* | hyperoxia | | | | slow | | 0.99 (0.43 – 3.15) | 1 | 11 |
|  | normoxia | | | | slow | | 0.80 (0.45 – 2.15) | 1 | 13 |
| sand goby, *Pomatoschistus minutus* | hyperoxia | | | | slow | | 1.58 (1.31 – 2.18) | 1 | 15 |
|  | normoxia | | | | slow | | 1.68 (0.71 – 2.57) | 1 | 8 |
| zebrafish, *Danio rerio* | hyperoxia | | | | slow | | 0.26 (0.12 – 0.39) | 2 | 29 |
|  | normoxia | | | | slow | | 0.25 (0.11 – 0.43) | 2 | 31 |
| Humbug damselfish, *Dascyllus aruanus* | hyperoxia | | | | slow | | 1.32 (0.13 – 5.63) | 2 | 30 |
|  | normoxia | | | | slow | | 1.41 (0.13 – 4.57) | 2 | 28 |
| Polynesian anemonefish, *Amphiprion maohiensis* | hyperoxia | | | | slow | | 1.06 (0.59 – 2.46) | 1 | 15 |
|  | normoxia | | | | slow | | 1.26 (0.64 – 2.46) | 1 | 15 |
| brown shrimp | | hyperoxia | | slow | | 0.68 (0.24 – 1.24) | | 1 | 19 |
|  |  | normoxia | | slow | | 0.58 (0.38 – 0.88) | | 1 | 16 |
| Baltic prawn | | | hyperoxia | slow | | 1.04 (0.53 – 1.78) | | 1 | 17 |
|  |  |  | normoxia | slow | | 1.66 (0.57 – 3.15) | | 1 | 17 |
|  |  | | | |  | | **Total** | **147** | **1451** |
