# Supplementary material for: Oxygen supersaturation has negligible effects on warming tolerance across diverse aquatic ectotherms
Source: PLoS Biol. 2025 Nov 4;23(11):e3003413. doi: 10.1371/journal.pbio.3003413 (PMC12585006; doi:10.1371/journal.pbio.3003413)
Supplement: S3 Table — The mass covariate (log transformed) was removed if not significant (P > 0.05) in the final model, but we give the mass coefficient estimate and P values from the full model in those cases where it was not significant. The bottom five models are based on linear mixed effects models with random intercepts and slopes, except for the “fish” model and the “slow warming” model, which were fit better using random intercepts only (based on comparison of AIC values and log-likelihood tests). (DOCX) [file pbio.3003413.s003.docx]

**Supplementary Information** **for**
*Oxygen supersaturation has negligible effects on warming tolerance across diverse aquatic ectotherms*

**S3 Table.** Model estimates for normoxia (intercept) and for the effects of hyperoxia for each of the 24 experiments modeled with separate linear models for each species. The mass covariate (log transformed) was removed if not significant (*P* > 0.05) in the final model, but we give the mass coefficient estimate and *P* values from the full model in those cases where it was not significant. The bottom five models are based on linear mixed effects models with random intercepts and slopes, except for the ‘fish’ model and the ‘slow warming’ model which were fit better using random intercepts only (based on comparison of AIC values and log-likelihood tests).

| **Species** | **Rate of warming (°C/hour)** | **Intercept (Warming tolerance at normoxia,° C) ± S.E.** | **Treatment coefficient (effect of hyperoxia, ° C) ± S.E.** | ***P*** | **Coefficient: log10 mass (g) ± S.E.** | ***P*** |
| --- | --- | --- | --- | --- | --- | --- |
| bluntnose minnow | 18 | 34.04 ± 0.27 | -0.02 ± 0.38 | 0.96 | 1.82 ± 0.69 | 0.01 |
| bluegill | 18 | 33.68 ± 0.25 | 0.38 ± 0.31 | 0.22 | 2.03 ± 0.62 | 0.002 |
| brook trout | 18 | 27.94 ± 0.17 | 0.16 ± 0.07 | 0.02 | 0.71 ± 0.22 | 0.002 |
| three-spined stickleback | 18 | 32.78 ± 0.08 | 0.14 ± 0.12 | 0.237 | -0.61 ± 0.47 | 0.19 |
| lesser pipefish | 18 | 31.74 ± 0.07 | 0.05 ± 0.09 | 0.60 | -0.31 ± 0.30 | 0.31 |
| European flounder | 18 | 30.73 ± 0.10 | 0.10 ± 0.10 | 0.35 | 1.55 ± 0.25 | <0.001 |
| sand goby | 18 | 29.27 ± 0.12 | 0.20 ± 0.17 | 0.24 | -1.05 ± 0.54 | 0.06 |
| zebrafish | 18 | 42.02 ± 0.09 | -0.14 ± 0.12 | 0.24 | 1.06 ± 0.55 | 0.06 |
| humbug damselfish (2023) | 18 | 39.27 ± 0.05 | -0.23 ± 0.07 | 0.002 | 0.10 ± 0.10 | 0.31 |
| humbug damselfish  experiment 2 (2024) | 18 | 38.88 ± 0.08 | 0.10 ± 0.11 | 0.36 | 0.05 ± 0.10 | 0.66 |
| Polynesian anemonefish | 18 | 38.30 ± 0.13 | 0.29 ± 0.17 | 0.10 | -0.45 ± 0.58 | 0.44 |
| brown shrimp (2022) | 18 | 30.83 ± 0.24 | 1.06 ± 0.34 | 0.002 | -0.63 ± 0.48 | 0.19 |
| brown shrimp experiment 2 (2024) | 18 | 33.72 ± 0.14 | 0.21 ± 0.19 | 0.28 | -0.70 ± 0.69 | 0.31 |
| Baltic prawn | 18 | 34.34 ± 0.06 | 0.27 ± 0.09 | 0.003 | -0.95 ± 0.22 | <0.001 |
| green crab | 18 | 34.92 ± 0.08 | -0.20 ± 0.11 | 0.07 | 0.36 ± 0.21 | 0.10 |
| rusty crayfish | 18 | 36.47 ± 0.16 | 0.44 ± 0.23 | 0.06 | -0.45 ± 0.30 | 0.14 |
| sand goby | 1 | 31.19 ± 0.38 | -0.19 ± 0.34 | 0.58 | -4.71 ± 1.40 | 0.003 |
| European flounder | 1 | 29.62 ± 0.08 | 0.16 ± 0.11 | 0.18 | 0.41 ± 0.25 | 0.11 |
| brook trout | 1 | 28.41 ± 0.11 | 0.16 ± 0.07 | 0.06 | 1.06 ± 0.37 | 0.008 |
| zebrafish | 1 | 41.25 ± 0.04 | 0.05 ± 0.05 | 0.36 | -0.09 ± 0.21 | 0.65 |
| Polynesian anemonefish | 1 | 37.04 ± 0.05 | 0.27 ± 0.07 | <0.001 | 0.07 ± 0.20 | 0.73 |
| humbug damselfish | 1 | 38.05 ± 0.05 | 0.19 ± 0.07 | 0.007 | -0.05 ± 0.08 | 0.46 |
| brown shrimp | 1 | 33.27 ± 0.19 | 0.13 ± 0.26 | 0.62 | -1.43 ± 0.93 | 0.13 |
| Baltic prawn | 1 | 34.97 ± 0.10 | 0.79 ± 0.15 | <0.001 | 0.08 ± 0.47 | 0.87 |
| Fish | 1 and 18 | 34.27 ± 1.32 | 0.11 ± 0.05 | 0.03 |  |  |
| Crustaceans | 1 and 18 | 34.06 ± 0.67 | 0.39 ± 0.16 | 0.01 |  |  |
| Fast warming | 18 | 34.27 ± 0.97 | 0.18 ± 0.08 | 0.02 |  |  |
| Slow warming | 1 | 34.07 ± 1.63 | 0.20 ± 0.05 | <0.001 |  |  |
| Overall | 1 and 18 | 34.20 ± 0.82 | 0.19 ± 0.06 | 0.002 |  |  |
