# Supplementary material for: Oxygen supersaturation has negligible effects on warming tolerance across diverse aquatic ectotherms
Source: PLoS Biol. 2025 Nov 4;23(11):e3003413. doi: 10.1371/journal.pbio.3003413 (PMC12585006; doi:10.1371/journal.pbio.3003413)
Supplement: S4 Table — The F and P values are from ANOVAs testing for differences among replicate CTmax trials. The CTmax mean range refers to the difference between the highest and lowest mean within-trial CTmax values. The data are visualized in S3 Fig. (DOCX) [file pbio.3003413.s004.docx]

**Supplementary Information** **for**
*Oxygen supersaturation has negligible effects on warming tolerance across diverse aquatic ectotherms*

**Table S4.** Statistics describing variation in CT_max_ among fast-warming (0.3°C min^-1^) replicate trials within a species and treatment (3-5 replicate trials per group, *ca*. 7-10 animals per replicate, see S2 Table for sample sizes). The *F* and *P* values are from ANOVAs testing for differences among replicate CT_max_ trials. The CT_max_ mean range refers to the difference between the highest and lowest mean within-trial CT_max_ value. The data are visualized in S3 Figure.

| Species | Treatment | *F* value | *P* value | Lowest mean CT_max_ | Highest mean CT_max_ | CT_max_ mean range (°C) |
| --- | --- | --- | --- | --- | --- | --- |
| bluntnose minnow | hyperoxia | 5.75 | 0 | 32.2 | 34.9 | 2.68 |
| bluntnose minnow | normoxia | 15.82 | 0 | 32.3 | 35.2 | 2.92 |
| brook trout | hyperoxia | 0.09 | 0.91 | 28.6 | 28.6 | 0.05 |
| brook trout | normoxia | 0.79 | 0.51 | 28.4 | 28.6 | 0.19 |
| Polynesian anemonefish | hyperoxia | 3.55 | 0.03 | 37.9 | 39.0 | 1.10 |
| Polynesian anemonefish | normoxia | 3.34 | 0.03 | 38.0 | 38.7 | 0.71 |
| rusty crayfish | hyperoxia | 0.56 | 0.64 | 36.7 | 37.1 | 0.44 |
| rusty crayfish | normoxia | 1.31 | 0.29 | 36.1 | 37.0 | 0.89 |
| European flounder | hyperoxia | 10.77 | 0 | 29.7 | 30.9 | 1.14 |
| European flounder | normoxia | 1.35 | 0.27 | 30.2 | 30.6 | 0.39 |
| green crab | hyperoxia | 0.52 | 0.67 | 34.6 | 34.8 | 0.17 |
| green crab | normoxia | 13.56 | 0 | 34.3 | 35.2 | 0.85 |
| humbug damselfish experiment 1 (2023) | hyperoxia | 0.50 | 0.73 | 38.9 | 39.2 | 0.23 |
| humbug damselfish experiment 1(2023) | normoxia | 2.45 | 0.08 | 39.1 | 39.5 | 0.39 |
| humbug damselfish experiment 2 (2024) | hyperoxia | 1.36 | 0.28 | 38.8 | 39.1 | 0.33 |
| humbug damselfish experiment 2 (2024) | normoxia | 1.23 | 0.31 | 38.8 | 39.0 | 0.24 |
| bluegill | hyperoxia | 1.15 | 0.34 | 33.2 | 34.1 | 0.91 |
| bluegill | normoxia | 0.69 | 0.57 | 32.7 | 33.6 | 0.94 |
| lesser pipefish | hyperoxia | 1.64 | 0.2 | 31.7 | 32.0 | 0.37 |
| lesser pipefish | normoxia | 4.59 | 0.01 | 31.4 | 32.0 | 0.56 |
| Baltic prawn | hyperoxia | 1.66 | 0.17 | 34.3 | 34.8 | 0.49 |
| Baltic prawn | normoxia | 20.6 | 0 | 33.6 | 34.9 | 1.38 |
| sand goby | hyperoxia | 9.41 | 0 | 28.8 | 29.9 | 1.11 |
| sand goby | normoxia | 1.07 | 0.38 | 29.0 | 29.5 | 0.52 |
| brown shrimp experiment 1 (2022) | hyperoxia | 21.9 | 0 | 30.9 | 33.1 | 2.23 |
| brown shrimp experiment 1 (2022) | normoxia | 12.6 | 0 | 29.4 | 32.5 | 3.16 |
| brown shrimp experiment 2 (2024) | hyperoxia | 3.09 | 0 | 33.5 | 34.3 | 0.74 |
| brown shrimp experiment 2 (2024) | normoxia | 13.4 | 0 | 33.0 | 34.3 | 1.27 |
| threespine stickleback | hyperoxia | 1.71 | 0.18 | 32.5 | 33.1 | 0.52 |
| threespine stickleback | normoxia | 0.93 | 0.44 | 32.7 | 33.0 | 0.35 |
| zebrafish | hyperoxia | 1.20 | 0.33 | 41.5 | 42.0 | 0.58 |
| zebrafish | normoxia | 2.27 | 0.09 | 41.8 | 42.2 | 0.45 |
