# Supplementary material for: Oxygen supersaturation has negligible effects on warming tolerance across diverse aquatic ectotherms
Source: PLoS Biol. 2025 Nov 4;23(11):e3003413. doi: 10.1371/journal.pbio.3003413 (PMC12585006; doi:10.1371/journal.pbio.3003413)
Supplement: S1 Fig — Animals from the normoxia treatment are shown in blue circles, hyperoxia in yellow diamonds. Linear relationships are shown where they were statistically significant (P < 0.01, see S3 Table). The 16 top panels are from the fast-warming trials (0.3°C min−1), the bottom eight panels shaded in blue are the slow-warming (1°C h−1) trials. The species are as follows: (i) bluntnose minnow Pimephalus notatus, (ii) bluegill Lepomis macrochirus, (iii) brook trout Salvelinus fontinalis, (iv) three-spined stickleback Gasterosteus aculeatus, (v) lesser pipefish Syngnathus rostellatus (vi), European flounder Platichthys flesus (vii), sand goby Pomatoschistus minutus, (viii) zebrafish Danio rerio, (ix) humbug damselfish Dascyllus aruanus experiment 1 (2023), (x) humbug damselfish experiment 2 (2024), (xi) Polynesian anenomefish Amphiprion maohiensis, (xii) green crab Carcinus maenas, (xiii) rusty crayfish Faxonius rusticus, (xiv) brown shrimp Crangon crangon experiment 1 (2022), (xv) brown shrimp C. crangon experiment 2 (2024), (xvi) Baltic prawn Palaemon adspersus, (xvii) sand goby, (xviii) European flounder, (xix) brook trout, (xx) zebrafish, (xxi) humbug damselfish, (xxii) Polynesian anenomefish, (xxiii) Baltic prawn, (xxiv) brown shrimp. (DOCX) [file pbio.3003413.s005.docx]

**Supplementary Information** **for**
*Oxygen supersaturation has negligible effects on warming tolerance across diverse aquatic ectotherms*


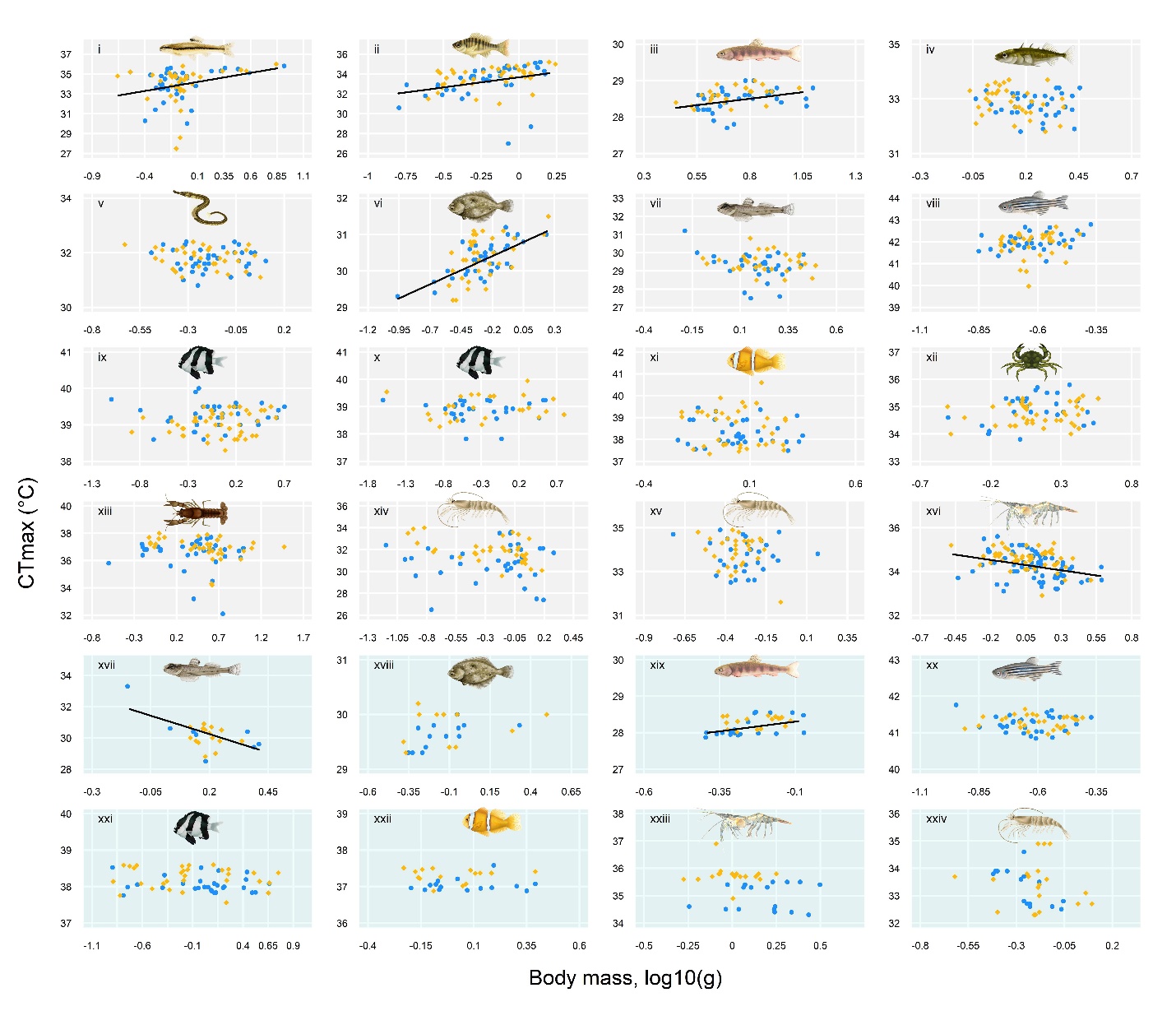


**S1 Figure.** Temperature at which loss of motor function occurred (CT_max_) in 24 experiments including 14 species of aquatic ectotherms, as a function of body mass (log_10_-transformed, as in our statistics). Animals from the normoxia treatment are shown in blue circles, hyperoxia in yellow diamonds. Linear relationships are shown where they were statistically significant (*P* < 0.01, see S3 Table). The 16 top panels are from the fast-warming trials (0.3°C min^-1^), the bottom eight panels shaded in blue are the slow-warming (1°C h^-1^) trials. The species are as follows: (i) bluntnose minnow *Pimephalus notatus*, (ii) bluegill *Lepomis macrochirus*, (iii) brook trout *Salvelinus fontinalis*, (iv) three-spined stickleback *Gasterosteus aculeatus*, (v) lesser pipefish *Syngnathus rostellatus* (vi), European flounder *Platichthys flesus* (vii), sand goby *Pomatoschistus minutus*, (viii) zebrafish *Danio rerio*, (ix) humbug damselfish *Dascyllus aruanus* experiment 1 (2023), (x) humbug damselfish experiment 2 (2024), (xi) Polynesian anemonefish *Amphiprion maohiensis*, (xii) green crab *Carcinus maenas*, (xiii) rusty crayfish *Faxonius rusticus*, (xiv) brown shrimp *Crangon crangon* experiment 1 (2022), (xv) brown shrimp *Crangon crangon* experiment 2 (2024), (xvi) Baltic prawn *Palaemon adspersus*, (xvii) sand goby, (xviii) European flounder, (xix) brook trout, (xx) zebrafish, (xxi) humbug damselfish, (xxii) Polynesian anemonefish, (xxiii) Baltic prawn, (xxiv) brown shrimp.
