# Supplementary material for: Oxygen supersaturation has negligible effects on warming tolerance across diverse aquatic ectotherms
Source: PLoS Biol. 2025 Nov 4;23(11):e3003413. doi: 10.1371/journal.pbio.3003413 (PMC12585006; doi:10.1371/journal.pbio.3003413)
Supplement: S2 Fig — In short, 9−10 seagrass Zostera marina meadows, where all the above-listed species were found, within 10 km of Kristineberg Marine Station (58.24965 N, 11.44585 E), were sampled using a handheld oximeter at 1 m depth for temperature, salinity, and dissolved oxygen in June, September, and October 2022. The oxygen saturation point was then calculated using the o2.at.sat function in the LakeMetabolizer (Winslow and colleagues, 2016, https://doi.org/10.1080/IW-6.4.883) package with the “garcia-benson” model applied to the data. From this, the oxygen saturation level of each site and date was calculated as %O2 = O2/O2′ × 100, where O2 was the dissolved oxygen in the sample in mg L−1 and O2′ was the oxygen solubility for each measurement of salinity and temperature. Blue circles show the calculated oxygen saturation. Green triangles show the corresponding temperature (shown on the right y-axis) measured at each site and date. Lines show the average value for all measurement points and months. (DOCX) [file pbio.3003413.s006.docx]

**Supplementary Information** **for**
*Oxygen supersaturation has negligible effects on warming tolerance across diverse aquatic ectotherms*


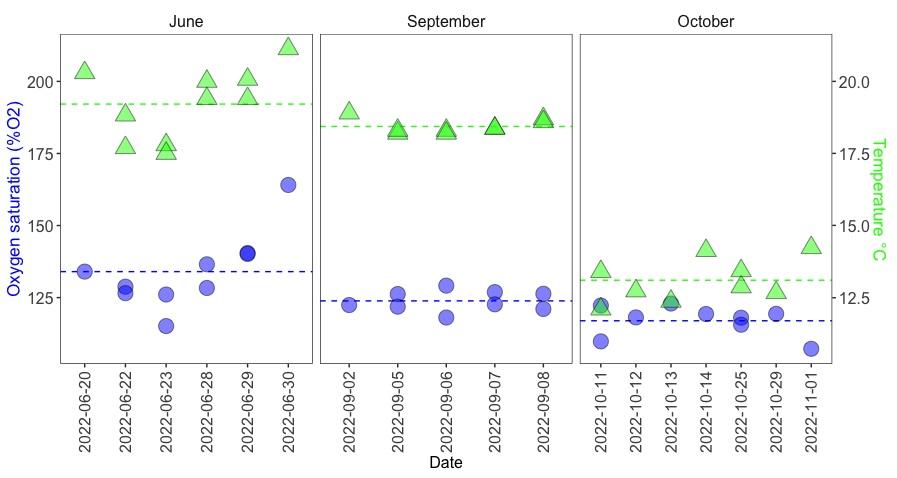


**S2 Figure.** Measurements of supersaturation in the area where the following species were collected for the study’s experiments: lesser pipefish *Syngnathus rostellatus*, three-spine stickleback *Gasterosteus aculeatus*, sand goby *Pomatoschistus minutus*, European flounder *Platichthys flesus*, green crab *Carcinus maenas* and brown shrimp *Crangon crangon.* In short, 9-10 seagrass *Zostera marina* meadows, where all the above listed species were found, within 10 km of Kristineberg Marine Station (58.24965 N, 11.44585 E), were sampled using a handheld oximeter at 1 m depth for temperature, salinity, and dissolved oxygen in June, September, and October 2022. The oxygen saturation point was then calculated using the o2.at.sat function in the LakeMetabolizer (Winslow et al., 2016, https://doi.org/10.1080/IW-6.4.883) package with the “garcia-benson” model applied to the data. From this, the oxygen saturation level of each site and date was calculated as %O_2_ = O2 / O2’ x 100, where O_2_ was the dissolved oxygen in the sample in mg L^-1^ and O_2_’ was the oxygen solubility for each measurement of salinity and temperature. Blue circles show the calculated oxygen saturation. Green triangles show the corresponding temperature (shown on the right y-axis) measured at each site and date. Lines show the average value for all measurement points and month.
