# Supplementary material for: Oxygen supersaturation has negligible effects on warming tolerance across diverse aquatic ectotherms
Source: PLoS Biol. 2025 Nov 4;23(11):e3003413. doi: 10.1371/journal.pbio.3003413 (PMC12585006; doi:10.1371/journal.pbio.3003413)
Supplement: S3 Fig — Sample sizes are given in S2 Table. a: bluntnose minnow, b: bluegill, c: brook trout, d: zebrafish, e: threespine stickleback, f: lesser pipefish, g: sand goby, h: European flounder, i: humbug damselfish experiment 1 (2023), j: humbug damselfish experiment 2 (2024), k: Polynesian anemonefish, l: brown shrimp experiment 1 (2022), m: brown shrimp experiment 2 (2024), n: green crab, o: rusty crayfish, p: Baltic prawn. See S1 Fig caption for scientific names. (DOCX) [file pbio.3003413.s007.docx]

**Supplementary Information** **for**
*Oxygen supersaturation has negligible effects on warming tolerance across diverse aquatic ectotherms*

**
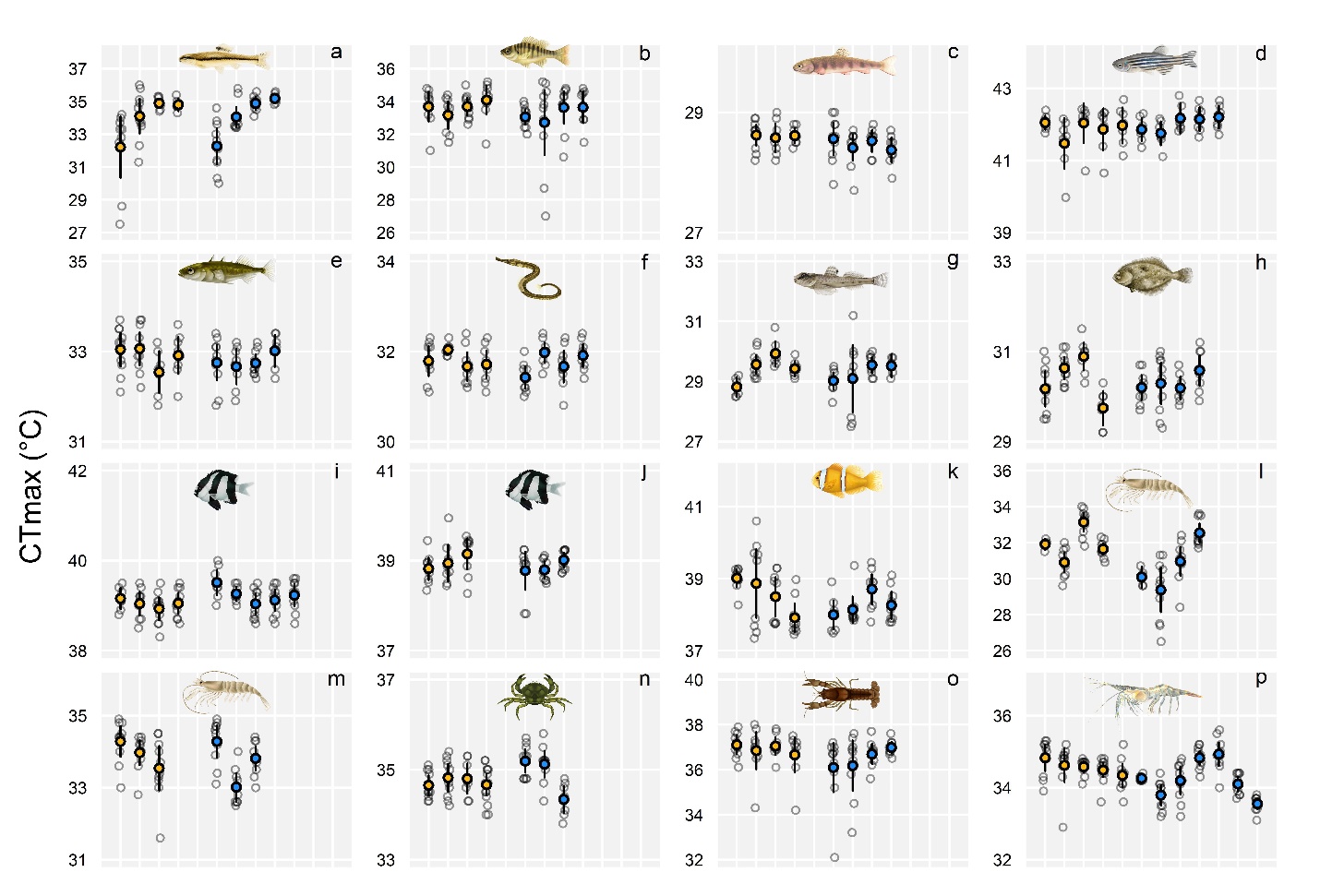
**

**S3 Figure**. CT_max_ data for fast-warming (0.3°C min^-1^) plotted separately by replicate trials, with individual data points shown and mean (yellow = hyperoxia, blue = normoxia) and 95% confidence intervals plotted for each group. Sample sizes are given in table S2. a: bluntnose minnow, b: bluegill, c: brook trout, d: zebrafish, e: threespine stickleback, f: lesser pipefish, g: sand goby, h: European flounder, i: humbug damselfish experiment 1 (2023), j: humbug damselfish experiment 2 (2024), k: Polynesian anemonefish, l: brown shrimp experiment 1 (2022), m: brown shrimp experiment 2 (2024), n: green crab, o: rusty crayfish, p: Baltic prawn. See Fig. S1 caption for scientific names.
