# Supplementary material for: Oxygen supersaturation has negligible effects on warming tolerance across diverse aquatic ectotherms
Source: PLoS Biol. 2025 Nov 4;23(11):e3003413. doi: 10.1371/journal.pbio.3003413 (PMC12585006; doi:10.1371/journal.pbio.3003413)
Supplement: S4 Fig — a: The arena we used for stickleback, zebrafish, lesser pipefish, sand goby, green crab, brown shrimp, and European flounder with a total water volume of 12 L. b: The arena we used for humbug damselfish and Polynesian anemonefish in 2024 with a water volume 8 L for fast-warming, 18 L for slow-warming; a similar arena was used in 2023 (humbug damselfish). c: the arena we used for brook trout, bluntnose minnow, rusty crayfish, and bluegill, with a water volume of 26 L. d: the arena (left = arena where the fish were confined, right = sump containing heaters, pumps, and air stones) that we used for the slow-warming sand goby and flatfish trials with a total water volume of 35 L. (DOCX) [file pbio.3003413.s008.docx]

**Supplementary Information** **for**
*Oxygen supersaturation has negligible effects on warming tolerance across diverse aquatic ectotherms*


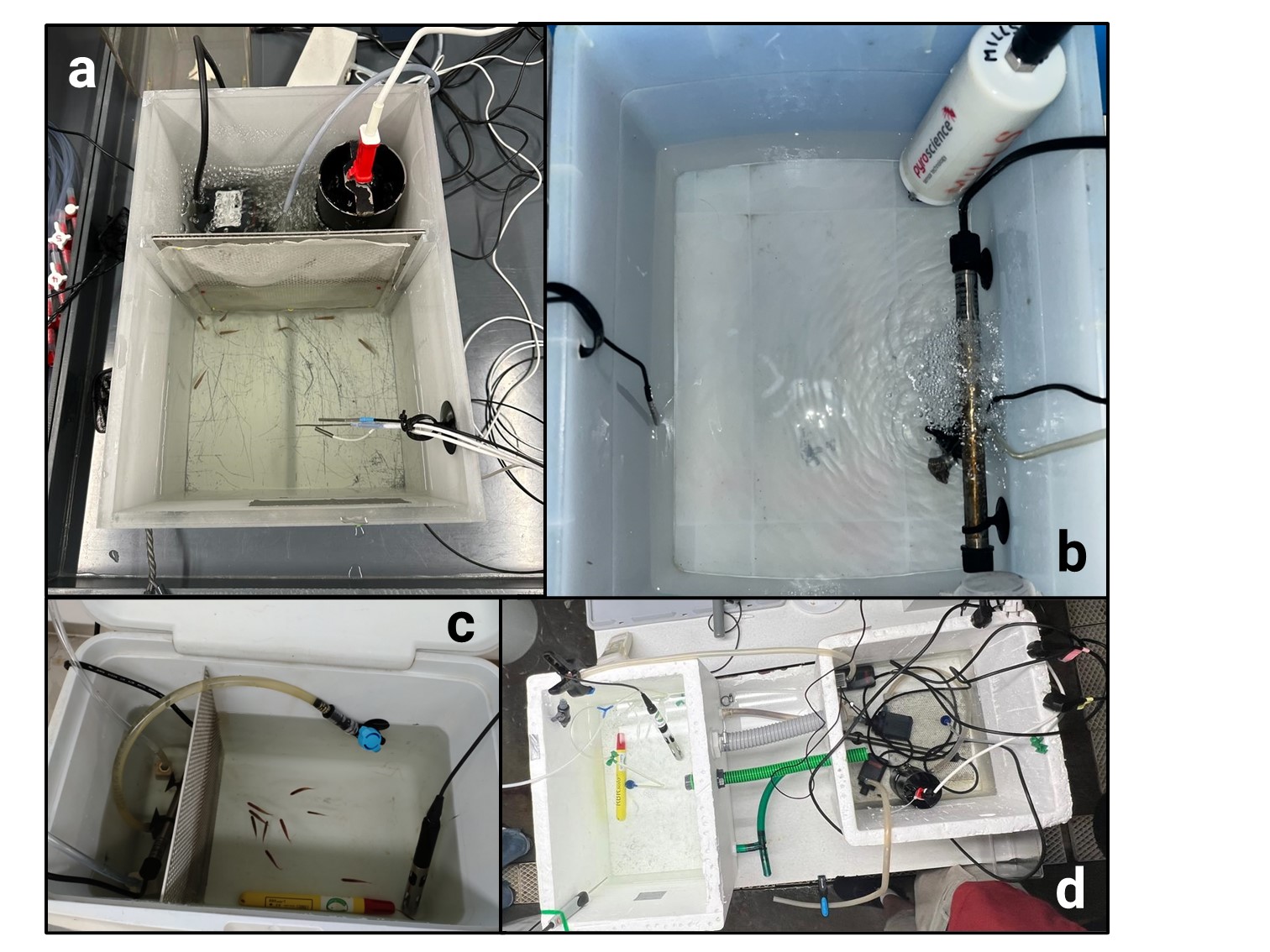


**S4 Figure.** Overhead photos of CT_max_ arenas we used. **a**: The arena we used for stickleback, zebrafish, lesser pipefish, sand goby, green crab, brown shrimp, and European flounder with a total water volume of 12 L. **b**: The arena we used for humbug damselfish and Polynesian anemonefish in 2024 with a water volume 8 L for fast-warming, 18 L for slow-warming; a similar arena was used in 2023 (humbug damselfish). **c**: the arena we used for brook trout, bluntnose minnow, rusty crayfish, and bluegill, with a water volume of 26 L. **d**: the arena (left = arena where the fish were confined, right = sump containing heaters, pumps, and air stones) that we used for the slow-warming sand goby and flatfish trials with a total water volume of 35 L.
